# Supplementary material for: Content-rich biological network constructed by mining PubMed abstracts
Source: BMC Bioinformatics. 2004 Oct 8;5:147. doi: 10.1186/1471-2105-5-147 (PMC528731; doi:10.1186/1471-2105-5-147)
Supplement: Additional File 5 — The original Chilibot query results of the term "long-term potentiation (LTP)" and 22 other terms, limiting the latest references analyzed to the years 1990, 1995, 2000, and 2004. [file 1471-2105-5-147-S5.bz2 › chilibotAdditionalFile5/ltp2004/html/PKA_CAMKII.html]

 


 **PKA** and **CAMKII** 
  
Found 84 abstracts in PubMed,  **30 abstracts were retrieved and analyzed**.  


---

 Search Google  |
 PDF files only 
|  EDU domain only 

---

**Interactive relationship** (e.g. stimulation, inhibition, etc)

- We concluded that serotonin, by activating 5 HT receptors, suppress glutamatergic signaling through the inhibition of  **CaMKII** , which is achieved by the inhibition of  **PKA**  and ensuing activation of PP1.  Ref: 12149253 J Biol Chem, 2002

- :-)

  **Parallel relationship** (e.g. studied together, co-existance, homology, etc.)

  - MAPK cascade interacts extensively with other protein kinases such as maturation promoting factor MPF, protein kinase A  **PKA** , protein kinase C PKC, and calmodulin dependent protein kinase II  **CaMKII**  as well as protein phosphatases in oocyte meiotic cell cycle regulation.  Ref: 14613897 Biol ReprodBiol Reprod, 2002
  - The present study assessed whether such changes depended on protein kinase A  **PKA**  and calcium calmodulin dependent protein kinase II  [ **CAMKII** ]   **CaMKII** .  Ref: 14743449 J Neurosci Res, 2004
  - These experiments reveal, unexpectedly, the existence of two parallel kinase pathways, one involving  **CaMKII**  and the other  **PKA**  and PKC, either of which can fully support the induction of LTP, at this stage of development.  Ref: 14529720 Neuropharmacology, 2003
  - The lack of Thr 17 phosphorylation at low isoproterenol concentrations might therefore be attributed to a level of  **PKA**  activity insufficient to increase Ca i to activate the  **CaMKII**  system and or to inhibit the phosphatase that dephosphorylates PLB  Ref: 12355181 Pflugers Arch, 2002
  - Inhibition of either PP 1 or calcineurin abolished RP impairment by  **PKA**  inhibition, but not that by  **CaMKII**  inhibition.  Ref: 12019316 J Neurosci, 2002
  - MEKK and  **CaMKII** ... increased promoter activity 4.5 and 1.5 fold, respectively, in NB2A cells.  Ref: 12421350 J Neurochem, 2002
  - Whereas with 30 300 nM isoproterenol, phosphorylation of Thr 17, the calcium calmodulin dependent protein kinase II  **CaMKII**  site and Ser 16, the protein kinase A  **PKA**  site, contributed approximately 50% each to PLB phosphorylation, and both participated in the relaxant action of isoproterenol, at lower a level of beta adrenoceptor stimulation isoproterenol 0.3 3 nM, both effects were exclusively due to Ser 16 phosphorylation.  Ref: 12355181 Pflugers Arch, 2002
  - The role of NMDA glutamate receptors,  **PKA** , MAPK, and  **CAMKII**  in the hippocampus in extinction of conditioned fear.  Ref: 12625457 Hippocampus, 2003
  - The potentiation was inhibited by GF109203X, a selective inhibitor of protein kinase C PKC... a selective inhibitor of  **CaMKII** , or H 89, a selective inhibitor of protein kinase A  **PKA** .  Ref: 14499485 Brain Res Mol Brain Res, 2003
  - Four protein kinase cascades have garnered considerable attention in the study of information storage at both the synaptic and behavioral levels Ca phospholipid dependent protein kinase PKC, Ca calmodulin dependent protein kinase II  **CaMKII** , cAMP dependent protein kinase  **PKA** , and extracellular signal regulated kinase ERK.  Ref: 11954557 Neuroscientist, 2002
  - Next, we utilized activators and inhibitors of protein kinase A  **PKA** , protein kinase C PKC, mitogen activated protein kinase kinase MAPKK and calcium calmodulin dependent protein kinase II  [ **CAMKII** ]   **CaMKII**  to determine which kinases might be involved in phosphorylating the CREB in PZHPV 7 cells.  Ref: 12899525 Recent Results Cancer Res, 2003
  - In contrast,  **PKA**  phosphorylation of GluR1 and the activity of calcium calmodulin dependent kinase II  **CaMKII**  were both necessary for receptor incorporation.  Ref: 12536214 Nat Neurosci, 2003
  - In contrast, the beta 1 AR proapoptotic effect is associated with non  **PKA**  dependent increases in intracellular calcium and  **CaMKII**  activity.  Ref: 12618516 J Clin Invest, 2003
  - Considerable evidences have shown that calcium calmodulin dependent protein kinase II  [ **CAMKII** ]   **CaMKII** , protein kinase A  **PKA**  and protein kinase C PKC are important for LTP in hippocampus.  Ref: 14586032 J NeurophysiolJ Neurophysiol, 2003
  - We confirmed that inhibition of calcium calmodulin dependent protein kinase II  **CaMKII**  or  **PKA**  suppresses RP.  Ref: 12019316 J Neurosci, 2002
  - Membrane bound hm mcH 1 R, whose conformation is closer to its physiological state than that of the immunoprecipitated receptor, was also phosphorylated by PKC,  **PKA** ,  **CaMKII**  and PKG.  Ref: 14685295 Methods Find Exp Clin Pharmacol, 2003
  - When hm mcH 1 R, which had been immunoprecipitated with anti c myc antibody from Sf9 cell membranes, was incubated with PKC, cAMP dependent protein kinase  **PKA** , calcium calmodulin dependent protein kinase II  [ **CAMKII** ]   **CaMKII**  or cGMP dependent protein kinase PKG, the immunoprecipitated receptor was phosphorylated by these kinases.  Ref: 14685295 Methods Find Exp Clin Pharmacol, 2003
  - These findings indicate that  **CaMKII**  constitutes a novel  **PKA**  independent linkage of beta 1 AR stimulation to cardiomyocyte apoptosis that has been implicated in the overall process of chronic heart failure.  Ref: 12618516 J Clin Invest, 2003
  - PLB can be phosphorylated at Ser16 by cAMP dependent protein kinase  **PKA**  and Thr17 by calcium calmodulin dependent protein kinase  **CaMKII**  during beta agonist stimulation.  Ref: 12479233 Basic Res Cardiol, 2002
  - A  **CaMKII**  inhibitor, KN 93, produced similar blocks, suggesting that the postablation plasticities in these nuclei depended on  **PKA**  or  **CaMKII** .  Ref: 13130509 J Neurosci Res, 2003
  - The expression patterns of cyclic AMP dependent protein kinase  **PKA**  and protein kinase C PKC... were similar to those of the forms of GluR1 phosphorylated at S845 and S831, respectively.  Ref: 12676339 Neurosci Lett, 2003
  - Thus, the postablation plasticities in these nuclei probably depended on  **PKA**  or  **CaMKII** .  Ref: 14743449 J Neurosci Res, 2004
  - Although a decrease in the phosphorylation of both PLB residues Ser16,  **PKA**  site, and Thr17,  **CaMKII**  site was previously reported, experiments from our laboratory failed to detect this decrease.  Ref: 14577598 Mol Cell Biochem, 2003
  - These data are the first demonstration of the phosphorylation of H 1 R by four protein kinases, i.e., PKC,  **PKA** ,  **CaMKII**  and PKG, and provide fundamental information to help us further understand the relationship between H 1 R phosphorylation and desensitization of this receptor.  Ref: 14685295 Methods Find Exp Clin Pharmacol, 2003
  - The recent availability of the PLB knockout mouse, in combination with mutagenesis and transgenic technologies, have provided excellent model systems for expression of each of the phosphorylation site specific PLB mutants in the heart and elucidation of the functional interplay between  **PKA**  and  **CaMKII**  dependent pathways of PLB phosphorylation.  Ref: 12479233 Basic Res Cardiol, 2002
  - In contrast, KN 93 elevated 14 C GABA release in the contralateral MNTB, suggesting a downregulatory action of  **CaMKII** , an action opposite to that of  **PKA** .  Ref: 14743449 J Neurosci Res, 2004
  - The Roles of  **CaMKII** ,  **PKA**  and PKC in the Induction and Maintenance of LTP of C Fiber Evoked Field Potentials in Rat Spinal Dorsal Horn.  Ref: 14586032 J NeurophysiolJ Neurophysiol, 2004
  - Thus, these studies revealed novel insights into the interdependence and physiological significance of  **PKA**  Ser16 and  **CaMKII**  Thr17 pathways of PLB phosphorylation during beta adrenergic stimulation in the mammalian heart.  Ref: 12479233 Basic Res Cardiol, 2002
  - In this study, we asked whether the calcium dependency of inhibitory depression was associated with the activation of calcium calmodulin dependent protein kinase II  [ **CAMKII** ]   **CaMKII** , protein kinase C PKC, and or cAMP dependent protein kinase A  **PKA** .  Ref: 12360581 J Neurobiol, 2002
  - Furthermore, activation of GABA B R inhibited  **CaMKII**  activation through  **PKA**  inhibition and PP 1 activity.  Ref: 12019316 J Neurosci, 2002
  - Specifically, the contributions of three kinases protein kinase A  **PKA** , calcium calmodulin dependent kinase II  **CaMKII** , and protein kinase C PKC are assessed during the consolidation phase of avoidance conditioning.  Ref: 11991759 Neurobiol Learn Mem, 2002
  - Phosphorylation of phospholamban is also increased specifically at the  **CaMKII**  but not at the  **PKA**  phosphorylation site.  Ref: 12676814 Circ Res, 2003
  - Thus, three key postsynaptic kinases,  **CaMKII** , PKC, and  **PKA** , participate in the activity dependent depression of inhibitory MNTB LSO synapses during postnatal development.  Ref: 12360581 J Neurobiol, 2002
  - These data eliminate roles for  **PKA**  and  **CaMKII**  as counteracting kinases.  Ref: 14742732 J PhysiolJ Physiol, 2002
  - In this study, we assessed whether such changes depended on protein kinase A  **PKA**  and calcium calmodulin dependent protein kinase II  [ **CAMKII** ]   **CaMKII** .  Ref: 13130509 J Neurosci Res, 2003
  - However, inhibition of  **CaMKII**  together with inhibition of either  **PKA**  or PKC fully blocked the induction of LTP.  Ref: 14529720 Neuropharmacology, 2003
  - These results indicate that EPSC enhancement may be attributable to a postsynaptic signaling cascade involving calcium,  **PKA** , and  **CaMKII** .  Ref: 12574415 J Neurosci, 2003
  - We report here that, unlike LTP in the more mature CA1 region of the hippocampus, LTP in neonatal rodent hippocampus < ; 9 postnatal days, < ; P9 requires cyclic AMP dependent protein kinase A  **PKA**  but not calcium calmodulin dependent protein kinase II  **CaMKII** .  Ref: 12469130 Nat Neurosci, 2003
  - In present work the roles of these three protein kinases in the induction and maintenance of LTP of C fiber evoked field potentials were evaluated by application of specific inhibitors of  **CaMKII**  KN 93 and AIP,  **PKA**  Rp CPT cAMPS and PKC chelerythrine and Go 6983 at the recording segments before and after LTP induction in Urethane anesthetized Sprague Dawley rats.  Ref: 14586032 J NeurophysiolJ Neurophysiol, 2003
  - These results suggest that activation of  **CaMKII** ,  **PKA**  and PKC may be crucial for the induction and the early phase but not for the late phase maintenance of the spinal LTP.  Ref: 14586032 J NeurophysiolJ Neurophysiol, 2003
  - Here we show that sustained beta 1 AR stimulation promotes cardiac myocyte apoptosis by activation of calcium calmodulin kinase II  **CaMKII** , independently of  **PKA**  signaling.  Ref: 12618516 J Clin Invest, 2003
  - The role of  **PKA** ,  **CaMKII** , and PKC in avoidance conditioning permissive or instructive ? .  Ref: 11991759 Neurobiol Learn Mem, 2002
